# Aggressive fluid management in the critically ill: Pro

**DOI:** 10.1186/s40560-019-0361-9

**Published:** 2019-02-02

**Authors:** Katsura Hayakawa

**Affiliations:** Department of Emergency and Critical Care Medicine, Saitama Red Cross Hospital, 1-5 Shintoshin, Chuo-ku, Saitama City, Saitama 330-8553 Japan

**Keywords:** Fluid therapy, Hypovolemia, Early goal-directed therapy, Shock

## Abstract

**Background:**

This review is a “Pro-Con” discussion about the optimal fluid volume in critically ill patients in the intensive care unit (ICU). This article argues that fluids should be aggressively managed in critically ill patients.

**Main body:**

In recent years, restrictive fluid management has been thought to be beneficial for critically ill patients. Thus, to investigate whether fluid volumes have actually been restricted in practice, fluid volumes were compared between those used in the early goal-directed therapy (EGDT) study by Rivers et al. performed in 2001 and those used in the Protocolized Care for Early Septic Shock (ProCESS), Australasian Resuscitation in Sepsis Evaluation (ARISE), and Protocolized Management in Sepsis (ProMISe) studies performed between 2014 and 2015. The later studies did not have lower total fluid volumes than those in the EGDT study. This finding shows that the importance of administering a sufficient fluid volume before admission to the ICU has become widely accepted.

Fluid management strategies for critically ill patients can be divided into the following four phases: rescue (or salvage), optimization, stabilization, and de-escalation. Fluid therapy administered within 6 h of presentation covers the rescue and optimization phases. Because hemodynamic instability is observed in these phases, sufficient fluid should be administered for lifesaving and organ rescue purposes. As a strategy, water may be removed during the hemodynamically stable later phase after sufficient fluid volumes were given during the hemodynamically instable early phase.

**Conclusions:**

Performing aggressive fluid management is important to infuse a sufficient fluid volume proactively during the hemodynamically instable early phase of a critical illness.

## Background

Fluid therapy is an important treatment method for patients in shock because it improves microvascular blood flow and increases cardiac output. However, some problems related to fluid overload in the intensive care unit (ICU) have been highlighted in recent years [1]. There is some concern about fluid therapy becoming restrictive.

This review is a “Pro-Con” discussion about the optimal fluid volume in critically ill patients in the ICU, and it argues that fluids should be aggressively managed in critically ill patients.

## Main body

According to the systematic review reported by Malbrain et al. in 2014, which included 11 randomized controlled trials and 24 observational studies, the water balance 1 week after ICU admission was 5.48 L lower in the restrictive fluid management group than in the liberal group. Mortality was also significantly lower in the restrictive group (odds ratio 0.42, 95% CI 0.32–0.55) [2]. While these studies showed that fluid volume was limited in cases that ultimately survived, no mention was made of how to prevent excessive fluid volume during the hemodynamically instable phases.

Fluid volumes were compared between the early goal-directed therapy (EGDT) study performed by Rivers et al. in 2001 [3] and the Protocolized Care for Early Septic Shock (ProCESS) [4], Australasian Resuscitation in Sepsis Evaluation (ARISE) [5], and Protocolized Management in Sepsis (ProMISe) [6] studies performed between 2014 and 2015 to investigate whether fluid volume has actually become restricted (Table 1). Initially, the three studies in 2014 and 2015 seemed to show that the total intravenous fluid volume administered between 0 and 6 h after presentation was reduced compared with the volume reported in the 2001 study. However, approximately 2000 mL of fluid had already been administered between presentation at the emergency department and randomization into the studies (given as the baseline or prerandomization amount). When this fluid is added to the fluid volume administered between 0 and 6 h after presentation, the total fluid volume administered within 6 h does not decrease compared with the volume reported in the 2001 study by Rivers et al. This result shows that the importance of administering sufficient fluid before admission to the ICU has become widely accepted. The recommendation of 30 mL/kg as an initial fluid volume was subsequently set in the 2016 Surviving Sepsis Campaign Guidelines (SSCG) [7]. This recommendation is based on the results of the average volume of fluid prior to randomization given in the ProCESS and ARISE trials. According to this guideline, clinicians enable more precise determinations regarding the hemodynamic status of the patient by fixing the initial fluid volume.

**Table 1:** Comparisons of total intravenous fluid and vasopressor use of researches by Rivers et al. in 2001, and the ProCESS, ARISE, and ProMISe in 2014 to 2015

	Total intravenous fluid (mL)				Vasopressors use (%)			
	Baseline (pre-randomization)	0–6 h	6–72 h	0–72 h	Baseline (pre-randomization)	0–6 h	6–72 h	0–72 h
Rivers E, et al (2001) [3]								
Standard therapy (*n* = 133)	–	3499 ± 2438	10,602 ± 6216	13,358 ± 7729	–	30.3	42.9	51.3
EGDT (*n* = 130)	–	4981 ± 2984	8625 ± 5162	13,443 ± 6390	–	27.4	29.1	36.8
ProCESS (2014) [4]								
Protocol-based EGDT (*n* = 439)	2245 ± 1472	2805 ± 1957	4458 ± 3878	7253 ± 4605	19.1	54.9	47.6	60.4
Protocol-based standard (*n* = 446)	2226 ± 1363	3285 ± 1743	4918 ± 4308	8193 ± 4989	16.8	52.2	46.6	61.2
Usual care (*n* = 456)	2083 ± 1405	2279 ± 1881	4354 ± 3882	6633 ± 4560	15.1	44.1	43.2	53.7
ARISE (2014) [5]								
EGDT (*n* = 793)	2515 ± 1244	1964 ± 1415	4274 ± 3071	–	21.8	66.6	58.8	–
Usual care (*n* = 798)	2519 ± 1331	1713 ± 1401	4382 ± 3136	–	21.7	57.8	51.5	–
ProMISe (2015) [6]								
EGDT (*n* = 625)	1890 ± 1105	2226 ± 1443	4215 ± 3068	5946 ± 3740	2.4	53.3	57.9	60.5
Usual resuscitation (*n* = 626)	1965 ± 1149	2022 ± 1271	4366 ± 3114	5844 ± 3651	3.4	46.6	52.6	55.0

Plus-minus values are means ± SD

Baseline (pre-randomization) means pre-hospital fluids and fluids administered between presentation to the emergency department and randomization

The fluid management strategy for critically ill patients can be divided into four phases, namely, rescue (or salvage), optimization, stabilization, and de-escalation [8, 9]. Fluid therapy administered within 6 h of presentation covers the rescue and optimization phases. Because early effective fluid management can stabilize sepsis-induced tissue hypoperfusion, sufficient fluid should be administered for lifesaving and organ rescue purposes. According to a retrospective analysis by Murphy et al., patients achieving adequate initial fluid resuscitation (AIFR, defined as the administration of an initial fluid bolus of ≥ 20 mL/kg prior to the onset of therapy with vasopressors and the achievement of a central venous pressure of ≥ 8 mmHg within 6 h after the onset of therapy with vasopressors) had a lower in-hospital mortality rate than those who did not achieve AIFR (32.2% vs 60.6%, *p* < 0.001) [10].

The ProCESS [4], ARISE [5], and ProMISe [6] studies reported more vasopressor use than the study by Rivers et al. [3] within 0–6 h of presentation (Table 1). These data indicate that recently, vasopressors tend to be used more in the early phases. Hypovolemia may sometimes remain if a sufficient fluid volume is not administered, contributing to tissue hypoxia in septic patients. The status of global tissue hypoxia in the absence of hypotension is called “cryptic shock.” In the analysis by Puskarich et al., the mortality rate of cryptic shock is not significantly different from that of overt shock [11]. These studies suggest the need to screen and treat septic patients with cryptic shock and normotension. Even if blood pressure is normal based on vasopressor use, cryptic shock caused by hypovolemia may remain; thus, it is important to administer a sufficient fluid volume.

As a treatment strategy, water can be removed during the hemodynamically stable later phases (corresponding to the stabilization and de-escalation phases) after the administration of sufficient fluid volume in the hemodynamically instable early phases (corresponding to the rescue and optimization phases). The efficacy of this strategy has been demonstrated by the Fluid and Catheter Treatment Trial (FACTT) [12]. In the conservative group in which fluid therapy was restricted in the later phases, we found that the duration of mechanical ventilation was shorter than that in the liberal group. Notably, in this study, sufficient fluid was administered per protocol during the hemodynamically instable phases. Fluid therapy was only restricted in the phases during which the hemodynamics become stable, which is when a mean arterial pressure ≥ 60 mmHg could be maintained without the use of vasopressors.

## Conclusions

Proactively administering a sufficient fluid volume is important during the early phases (rescue and optimization phases) of a critical illness, during which the hemodynamics are instable; fluid administration should not be restricted in this stage. Restrictive fluid therapy should only be started in the later phases when the hemodynamics stabilize.

## Abbreviations

EGDT: Early goal-directed therapy
SSCG: Surviving Sepsis Campaign Guidelines
